# Supplementary material for: A systematic review of sample size estimation accuracy on power in malaria cluster randomised trials measuring epidemiological outcomes
Source: BMC Med Res Methodol. 2024 Oct 15;24:238. doi: 10.1186/s12874-024-02361-9 (PMC11476958; doi:10.1186/s12874-024-02361-9)
Supplement: Supplementary file 2 — Supplementary Material 2 [file 12874_2024_2361_MOESM2_ESM.docx]

**Additional file 3**: Shapiro-Wilk W tests of normality for predicted and observed prevalence, incidence and effect size distributions among included trials.
