# Supplementary material for: A systematic review of sample size estimation accuracy on power in malaria cluster randomised trials measuring epidemiological outcomes
Source: BMC Med Res Methodol. 2024 Oct 15;24:238. doi: 10.1186/s12874-024-02361-9 (PMC11476958; doi:10.1186/s12874-024-02361-9)
Supplement: Supplementary file 4 — Supplementary Material 4 [file 12874_2024_2361_MOESM4_ESM.docx]

**Additional file 4**: Intra-cluster correlation coefficients (ICC) used in malaria CRT sample size calculations included in this review.

| **Trial** | **Outcome** | **ICC** | **Method used to estimate ICC** |
| --- | --- | --- | --- |
| Agius 2020 | Incidence | 0.15 | Without data |
| Foy 2019 | Incidence | 0.02 | Without data |
| Homan 2016 | Incidence | 0.006 | With data |
| Manning 2018 | Incidence | 0.4 | Without data |
| Poespoprodjo 2021 | Incidence | 0.05 | Without data |
| Arzika 2019 | Prevalence | 0.056 | With data |
| Kone 2020 | Prevalence | 0.25 | With data |
| Minkawa 2021 | Prevalence | 0.053 | With data |
| Oldenburg 2018 | Prevalence | 0.075 | Without data |
| Von Seidlein 2019 | Prevalence | 0.07 | Without data |
